# False Alarm Effects in Early Warnings for Emergency Vehicles: Exploring Drivers’ Move-Over Behavior

**DOI:** 10.1177/00187208231216835

**Published:** 2023-11-29

**Authors:** Kajsa Weibull, Björn Lidestam, Erik Prytz

**Affiliations:** 1 25543 Swedish National Road and Transport Research Institute, Linköping, Sweden; 2 Department of Computer and Information Science, 4566 Linköping University, Linköping, Sweden; 3 Center for Disaster Medicine and Traumatology, and Department of Biomedical and Clinical Sciences, 4566 Linköping University, Linköping, Sweden

**Keywords:** driver behavior, intelligent vehicle systems, warning systems, expert–novice differences, warning compliance

## Abstract

**Objective:**

This study investigated drivers’ move-over behavior when receiving an Emergency Vehicle Approaching (EVA) warning. Furthermore, the possible effects of false alarms, driver experience, and modality on move-over behavior were explored.

**Background:**

EVA warnings are one solution to encourage drivers to move over for emergency vehicles in a safe and timely manner. EVA warnings are distributed based on the predicted path of the emergency vehicle causing a risk of false alarms. Previous EVA studies have suggested a difference between inexperienced and experienced drivers’ move-over behavior.

**Method:**

A driving simulator study was conducted with 110 participants, whereof 54 inexperienced and 56 experienced drivers. They were approached by an emergency vehicle three times. A control group received no EVA warnings, whereas the experimental groups received either true or false warnings, auditory or visual, 15 seconds before the emergency vehicle overtook them.

**Results:**

Drivers who received EVA warnings moved over more quickly for the emergency vehicle compared to the control group. Drivers moved over more quickly for each emergency vehicle interaction. False alarms impaired move-over behavior. No difference in driver behavior based on driver experience or modality was observed.

**Conclusion:**

EVA warnings positively affect drivers’ move-over behavior. However, false alarms can decrease drivers’ future willingness to comply with the warning.

**Application:**

The findings regarding measurements of delay can be used to optimize the design of future EVA systems. Moreover, this research should be used to further understand the effect of false alarms in in-car warnings.

## Introduction

Emergency driving is a demanding task. The emergency vehicle operator (EVO) is under pressure to arrive at the incident scene as soon as possible but must do so without imposing an undue risk to the people in or around the vehicle. The EVOs’ driving situation becomes more complex due to the interaction with other road users (Burke et al., 2001; Drucker et al., 2013; Hsiao et al., 2018; Koski & Sumanen, 2019).

To improve the safety of emergency vehicles (EVs), civilian drivers must move over in a safe and timely manner. Today, warning lights and sirens are used to warn drivers to move over when an EV is approaching. However, sirens can be difficult to localize and the distance to them is often overestimated (Caelli & Porter, 1980; Catchpole & Mckeown, 2007; Missikpode et al., 2018). In addition, modern vehicles have a high degree of soundproofing, which can lead to delayed detection of sirens. Delayed detection can result in an insufficient amount of time to plan and execute a safe move-over procedure.

One proposed method to support drivers when interacting with EVOs in traffic is to provide the drivers with an Emergency Vehicle Approaching (EVA) warning (Lenné et al., 2008). An EVA warning gives the driver an early in-car notification that an EV is approaching and encourages the driver to move over (Payre & Diels, 2020). Providing an EVA warning thus gives the driver more time to find a suitable way to move over. A handful of previous studies such as Lenné et al. (2008), Lidestam et al. (2020), Payre and Diels (2020), and Savolainen et al. (2010) have concluded that early warnings for approaching EVs can provide safety benefits.

Lidestam et al. (2020) suggested that inexperienced drivers may benefit more from EVA warnings, compared to experienced drivers. There are several reported differences between experienced and inexperienced drivers, such as risk assessments (Smith et al., 2009), visual scanning of surroundings (Deng et al., 2021), and crash involvement (Seacrist et al., 2016). Driver training rarely includes real-life interaction with an EV. Therefore, the first interaction where a move-over maneuver is required is likely to occur once the driver has their driver’s license. It is therefore important to examine if EVA warnings can assist inexperienced drivers in their interactions with EVs.

Effective warning systems must be detectable, reliable, and cause the receiver to take appropriate actions (Patterson, 1989). To form a reliable warning system, a warning must always be given only when it is needed. Failure to distribute a warning is denoted as a missed warning. The consequences of a missed warning could be major as it undermines the purpose of the warning system, which is to give a chance to prepare for imminent danger. However, minimizing the risk of missed alarms increases the risk of false alarms (Parasuraman et al., 1997). *The false alarm effect* is a theory anticipating that the credibility of the system is damaged when a threat fails to appear following a warning (Breznitz, 1984). A false alarm can cause the receiver to pay less attention to the next warning. As a result, the loss of credibility causes the receiver of the warning to take fewer protective measures the next time the warning is issued (Breznitz, 1984).

As more technology and warning systems are introduced in cars, the potential issues with false alarms for drivers have gained importance (Parasuraman et al., 1997). To the authors’ knowledge, no study has examined false alarm effects in the context of EVA warnings. However, in a study by Dingus et al. (1997), participants drove in traffic with various false alarm rates of collision warnings. The false alarm rates were 0–31%, 31–60%, or greater than 60%. The results showed that younger drivers (18–24 years old) were affected by the highest false alarm frequency, while the older drivers (60+ years old) did not change their behavior. A similar result was found by Ben-Yaacov et al. (2002), where naturalistic driving data was used to examine the effects of unreliable warning systems. There were no significant changes in driver behavior if the warning system had a reliability level of 60–95%. The findings of Dingus et al. (1997) and Ben-Yaacov et al. (2002) both indicate that false alarms do not alter driver behavior if they are in a minority, but if most warnings are false there is a risk of changed behavior. Even though the findings in Dingus et al. (1997) and Ben-Yaacov et al. (2002) may translate to EVA warnings, Lenné et al. (2008) suggested that EVA warnings have primarily a priming effect. This means that EVA causes drivers to move over more quickly once they see the EV. If that is the case, drivers will not move over if they are only given false EVA warnings.

The purpose of the present study is to explore EVA warnings in relation to false alarms and driver experience. The current study had four hypotheses. First, based on Lenné et al. (2008), Lidestam et al. (2020), Payre and Diels (2020), and Savolainen et al. (2010), that EVA should encourage drivers to move over more quickly, compared to drivers who do not receive EVA warnings. Second, experienced drivers should move over quicker compared to inexperienced drivers. Furthermore, based on Lidestam et al. (2020) inexperienced drivers should benefit more from EVA warnings compared to experienced drivers. Third, the current study included both visual and auditory EVA warnings (see Materials). Because no previous studies have explored modality in relation to EVA warnings, the null hypothesis was assumed. Fourth, based on the *false alarm effect*, previous false alarms should affect move-over behavior in future EVA warning situations. In an attempt to explore the hypotheses, a simulator study with inexperienced and experienced drivers was conducted.

## Method

This research complied with the American Psychological Association Code of Ethics and was approved by the Swedish Ethical Review Authority. Informed consent was provided by each participant.

### Participants

A total of 110 participants (50 women, 60 men) were included in the study. All participants had a valid driver’s license for regular passenger cars (category B in Sweden). They were divided into an inexperienced and an experienced driver group. An inexperienced driver had to be 18–24 years old and have had their driver’s license for a maximum of 5 years. If they had had their driver’s license for more than a year, their annual driving distance had to be less than 5000 km. A total of 54 inexperienced drivers aged 18–23 years (*M =* 20.5, *SD =* 1.53 yrs), whereof 29 males and 25 females, were included in this group. They had had their driver’s license for 0–5 years (*M* = 1.66, *SD* = 1.5 yrs) and drove 0–500 km per week (*M =* 65.5*, SD =* 96.7 km/week)*.* Of these, 11 had had their driver’s license for less than a year but with a driving distance exceeding 5000 km per year.

An experienced driver was required to have had their driver’s license for at least ten years, drive a minimum of 15,000 km annually, and be 40–65 years old. This group included 56 experienced drivers (31 men and 25 women) who were aged 40–61 years (*M* = 50.67, *SD* = 5.68 yrs). They received their driver’s license 10–42 years ago (*M* = 30.9, *SD* = 7.04 yrs) and drove 100–1250 km per week (*M* = 426.9, *SD* = 243.16 km/week). All participants were recruited through advertisements on social media and received a 200 SEK gift card for their participation.

### Materials

**Figure 1.**
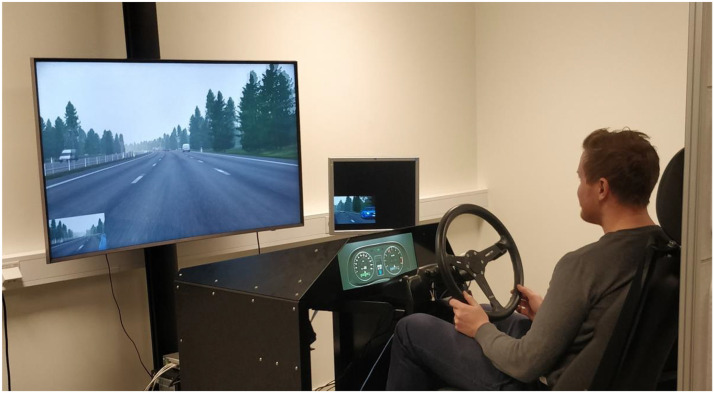
The simulator used in the present study.

A fixed-base simulator was used (Figure 1). A 55-inch screen with 1920 × 1080 resolution was placed about 1.5 m in front of the driver to display the simulated road and left rear-view mirror. One 19-inch screen, with a resolution of 1280 × 1024, was mounted to the right to display the right rear-view mirror. The screens were placed at an angle of about 170°. The background noise in the room was about 40 dBA. During driving the sound of the engine and surrounding traffic was about 70 dBA.

**Figure 2.**
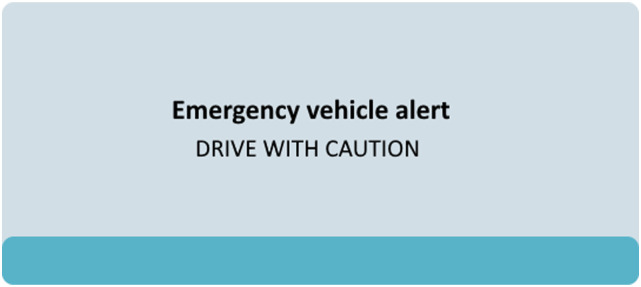
The visual EVA warning used in the present study.

The EVA message was either presented visually on the instrument cluster or through an auditory alert. The visual warning covered half of the instrument cluster and stated, “Emergency vehicle alert—drive with caution” (see Figure 2). For the auditory alert, a voice said at about 70 dBA “Varning! Utryckande fordon! Var god ge fri väg.” (“Warning! Emergency vehicle approaching! Please move over.”) The two warning alternatives were provided by two different companies and reflected their suggested design alternatives for EVA warnings. Both types of warnings were presented 15 seconds before the EV was expected to pass the participant’s vehicle and were presented until the EV overtook the participant’s vehicle.

It was possible to see the EV 21 seconds prior to it overtaking the participant’s vehicle. The siren sound was presented when the EV was 5 seconds from passing the participant’s vehicle. Blue lights, which were visible 5 seconds prior to overtaking, were presented by two LED strips placed behind the participant to create a reflective effect on the front screen. The intensity of the blue lights and the volume of the sirens increased as the EV approached the participant’s vehicle.

### Design

The overall experimental design was a 2 (Warning: Warning vs. Control [no warning]) × 2 (Verity: True warning vs. False warning) × 2 (Experience: Inexperienced vs. Experienced) × 2 (Modality: Auditory vs. Visual) × 3 (EV Event: 1st, 2nd, 3rd) split-plot factorial design. Warning, Verity, Experience, and Modality were between-group variables and EV Event within-group. All participants experienced three EV events. Each EV event could either entail no warning (for the control group), or a true or false warning (for the warning group). A true-warning condition involved an EVA warning (auditory or visual) 15 seconds before the EV caught up with the driver. In the false-warning condition, the participant received an EVA warning (auditory or visual), but no EV showed up. The conditions and number of participants per EV event are displayed in Table 1.

**Table 1.** Participant Distribution Over Experimental Conditions (C = Control, T = True EVA Warning, F = False EVA Warning).

	EV event		
	1	2	3
	C: 36	CC: 36	CCC: 20
			CCT: 16
	T: 42	TT: 26	TTT: 18
			TTF: 8
		TF: 16	TFT: 8
			TFF: 8
	F: 32	FF: 16	FFF: 8
			FFT: 8
		FT: 16	FTT: 8
			FTF: 8
*N* =	110	110	110

### Procedure

The participants were welcomed and informed about the procedure, their anonymity, and the infection control precautions (due to the ongoing COVID-19 pandemic). They then provided informed consent and responded to a preexperiment questionnaire. They were asked about their driving experience and demographics, such as age and gender. The participants were seated in the simulator and informed that they would drive with a cruise control set at 120 kph. The brake pedal was the only way to control the speed. Once the participant let go of the brake pedal, the car would automatically accelerate to 120 kph again. The participants were instructed to drive as they would normally do in real traffic and were informed that the experiment was regarding novel technical solutions. However, they did not receive specific information about the EVA warning systems.

The driving scenario began in the right-most lane with a one-minute practice drive at 30 kph to reduce the risk of simulator sickness. The main drive scenario consisted of a 30-min drive on a three-lane highway. The density of traffic was high in the right and middle lanes. Surrounding cars would on average maintain a lower speed than the participant, leading the participant to prefer the left-most lane.

The first EVA warning was presented after 11 minutes. If it was a true EV event either a police car or ambulance caught up with the participant’s vehicle. The EV was always driving in the left-most lane at 165 kph. The EV remained behind the participant’s vehicle with lights and sirens engaged until the participant moved over. The false warnings were presented for the same amount of time but ended without any approaching EV. The second and third EV events followed the same pattern, at minutes 19 and 27 into the scenario. In between the EV events, a civilian vehicle would approach the participant. These civilian vehicles maintained the same speed and behavior as the EVs. The civilian vehicles were introduced to ensure that not all vehicles approaching in the left-most lane were EVs.

The participants in the control condition were approached by EVs with the same timing but did not receive EVA warnings. After the drive, all participants were asked to fill out a postexperiment questionnaire where they were asked about prior experience with emergency vehicles. Before leaving, participants were informed about the purpose of the study and received a gift card.

### Data Analyses

Performance was measured by *move-over time*. The move-over time is the number of seconds it took for the participant to move over from the left lane after the EV was first visible. The participant was considered to have moved over when their lateral position was 2.5 m from the center position of the leftmost lane. This was the smallest lateral difference that allowed the EV to overtake the participant’s vehicle. The EV was visible 21 seconds before it would overtake the participant’s vehicle, assuming that both vehicles maintained constant speeds and that the participant moved over in time. The move-over time could thus be longer than 21 seconds, if the participant blocked the EV’s path. In the third event, one participant from the control group and one from the TTT group moved over before the emergency vehicle was visible. They were therefore removed from the analysis. None of the remaining participants had a move-over time of 6 seconds or less, which would have indicated that they moved over before the EVA warning was issued.

For the data analyses, only the control conditions and the true alarm events were used. This is because it is not necessarily the case that a shorter move-over time is the correct choice for the false alarm events, as this would mean that the participant moved over unnecessarily. For the true events, however, moving over is always the correct choice, and a shorter move-over time is preferable over a longer one. To investigate the effects of false alarms, the true alarm conditions in the second and third events were used to compare groups that had received only true alarms, a majority of true alarms, or a majority of false alarms.

To examine hypotheses one, regarding the effects of EVA warnings, and two, regarding the effects of experience, a 2 (Warning vs. Control) × 2 (Experience) × 3 (EV Event) split-plot factorial ANOVA, with Warning and Experience as between-groups variables and EV Event as repeated measures, was conducted. To examine hypothesis 3, regarding the effects of warning modality, a 2 (Modality) × 2 (Experience) × 3 (EV Event) split-plot factorial ANOVA was performed, with Modality as a between-subjects factor. This test only included drivers from the T group and no drivers from the control or false conditions. Pairwise comparisons were used to follow-up on the effects of EV Events.

To further examine the false alarm effect, in hypothesis 4, planned contrasts were performed for each EV event. Because the responses to false alarms could not be examined directly, no comparison between true and false warnings was performed in the first EV event. In the second EV event, two combinations (TT and FT) were explored, and in the third EV event four (TTT, FTT, TFT, and FFT; see also Table 1). Planned contrasts were performed per EV event (see Table 2). Planned contrast *A* compared the groups that only received true alarms with the groups that received at least one false alarm. Planned contrast *B* compared the groups that received a majority of true alarms with the groups that received a majority of false alarms.

**Table 2.** Planned Contrasts Explored for the 2nd and 3rd EV Events.

	2nd	3rd
A) Is there a difference between participants who receive false alarm(s) and participants who only receive true alarms?	×	×
B) Is there a difference in move-over time for those who receive a majority of false versus a majority of true alarms?		×

## Results

### Warning, Experience, and EV Event

**Figure 3.**
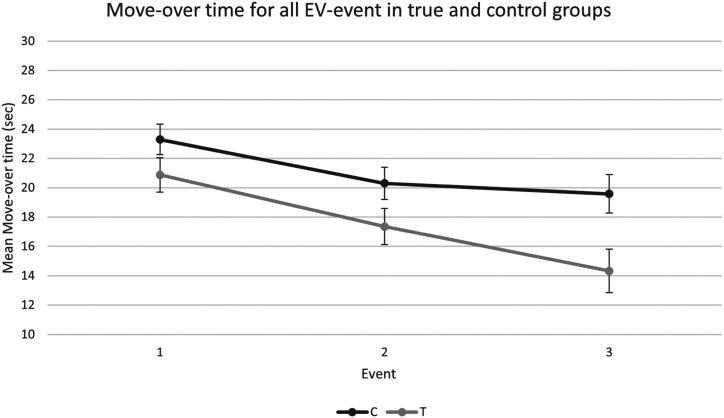
Mean move-over time (±*SE*) for true (T) and control (C) conditions in all EV events.

The 2 (Warning) × 3 (EV Event) × 2 (Experience) split-plot factorial ANOVA yielded a main effect of EV Event, *F*(2, 30) = 9.68, *p* < .001, $\eta_{p}^{2}$ = .392, such that the first event had the longest average move-over time (*M* = 22.38, *SD* = 4.60 sec), the second event the second longest (*M* = 18.87, *SD* = 4.85 sec), and the third the shortest move-over time (*M* = 17.14, *SD* = 6.08 sec). A main effect of Warning was shown, *F*(1, 31) = 8.45, *p* = .007, $\eta_{p}^{2}$ = .21, suggesting that drivers who received an EVA warning moved over more quickly (*M* = 17.52, *SD* = 5.39 sec), compared to drivers in the control group (*M* = 21.06, *SD* = 4.80 sec). Event 2 had a significantly lower move-over time compared to event 1, and event 3 significantly lower than event 1, indicating that drivers moved over more quickly in events 2 and 3, compared to event 1. Events 2 and 3 were not significantly different. The effects of Event and Warning are displayed in Figure 3.

No statistically significant difference was observed between the inexperienced (*M* = 19.44, *SD* = 4.80 sec) and experienced drivers (*M* = 19.13, *SD* = 5.39 sec), *F*(1, 31) = .06, *p* = .80. There were no significant interaction effects for Warning × Experience, *F*(1, 31) = .01, *p* = .91, Event × Warning, *F*(2, 30) = .87, *p* = .43, Event × Experience, *F*(2, 30) = .19, *p* = .82, or Event × Warning × Experience, *F*(2, 30) = .12, *p* = .30.

### Modality, Experience, and EV Event

The 3 (Event) × 2 (Modality) × 2 (Experience) split-plot factorial ANOVA showed a main effect of Event, *F*(2, 11) = 9.11, *p* = .005, $\eta_{p}^{2}$ = .62, such that the first event yielded the longest average move-over time (*M* = 21.82, *SD* = 5.91 sec), the second event the second longest (*M* = 16.99, *SD* = 4.35), and the third the shortest move-over time (*M* = 14.24, *SD* = 3.94 sec). The events were all statistically different from each other. There was no significant difference in move-over time, *F(*1, 12) = .12, *p* = .73, between the visual warning condition (*M* = 17.82, *SD* = 5 sec) and the auditory (*M* = 17.21, *SD* = 4.99 sec).

The difference in move-over time between inexperienced (*M* = 17.74, *SD* = 4.30 sec) and experienced drivers (*M* = 17.74, *SD* = 4.32 sec) did not result in a statistically significant difference, *F*(1, 12) = .07, *p* = .80. There were no significant interaction effects for Modality × Experience, *F*(1, 12) = .18, *p* = .68, Event × Experience, *F*(2, 11) = .73, *p* = .50, Event × Modality, *F*(2, 11) = 1.56, *p* = .25, or Event × Experience × Modality, *F*(2, 11) = .56, *p* = .59.

### False-Alarm Effect on Move-Over Time

To further examine hypothesis 4, planned contrasts were performed for events two and three. For the second EV event, only planned contrast A was performed. There was no significant difference in move-over time, *t*(37) *=* .49, *p* = .63, between the TT (*M* = 17.12, *SD* = 3.95 sec) and the FT groups (*M* = 17.87, *SD* = 2.22 sec).

To investigate planned contrast A (only true vs. false) in the third event, the TTT group was put in contrast to all groups that contained a false alarm. The TTT group had a shorter move-over time (*M* = 14.24, *SD* = 3.94 sec) compared to the groups that received at least one false alarm (*M* = 18.25, *SD* = 5.08 sec). The planned contrast showed a significant difference, *t*(35) = 2.75, *p* = .009, *d* = 2.68.

For planned contrast B (majority true vs. majority false), the groups with one false alarm (TFT, FTT) were contrasted with the group that received two false alarms before receiving a true alarm in the third event (FFT). The groups that received one false alarm had a shorter move-over time (*M* = 17.57, *SD* = 5.65 sec*)* compared to the group that received two false alarms (*M* = 19.81, *SD* = 3.26 sec). However, the difference was not significant *t*(35) = 1.89, *p =* .067.

## Discussion

The purpose of the present study was to explore EVA warnings in relation to false alarms and driver experience. The results show that drivers who receive an EVA warning move over faster compared to drivers who do not receive an EVA warning. The difference between the EVA and control group increased for each EV event. However, drivers from both the EVA and control group had a lowered move-over time for every event, indicating that there was a learning effect.

The present study included three EV interactions, which is perhaps not a common traffic situation. However, it is not an unlikely scenario if multiple emergency organizations are responding to a larger incident. The experimental design with multiple EV interactions was necessary to explore the possible false alarm effects. However, learning effects are unavoidable with reoccurring events in an experiment. In terms of ecological validity, it is likely that the first EV event in the current study best reflects how a driver who is unfamiliar with the EVA system would react. The participants in the present study were not informed about EVs or EVA warnings before partaking in the experiment. With an increasing number of driving support systems in vehicles, it is likely that drivers will not be aware of what functions they have in their cars (Harms et al., 2020). However, once they have experienced the EVA system, the second and third EV events in the current study could reflect how drivers adapt to a warning system that they are familiar with.

No differences between inexperienced and experienced drivers were found. Furthermore, there were no indications that inexperienced drivers would benefit more from EVA warnings. This is in contrast with previous studies (e.g., Dingus et al., 1997; Lidestam et al., 2020) that suggested that their behavior may differ. It may be that the difference is not prominent in a warning-response situation but rather when faced with risk assessment (Smith et al., 2009), or more demanding traffic situations (Deng et al., 2021) than in the current study. Furthermore, it could be that experienced drivers would manage an EV situation in real traffic better than inexperienced drivers. However, young, inexperienced drivers might be more familiar with technology and therefore more comfortable with driving in the simulator. The experience of how to handle an EV situation and the experience of handling simulators may counterbalance each other. Therefore, future studies should explore driver experience in a naturalistic road environment.

Both auditory and visual warnings were included in the present study. The results of the current study did not indicate a difference between the two modalities. However, the modalities were too different to make assumptions based on the findings of the present study. The auditory warning held an instruction while the visual warning only encouraged the driver to drive with caution. Finally, the auditory warning was in Swedish, the native language of all participants, while the visual warning was in English. This is a limitation of the current study and future studies should include warnings that allow comparisons between different modalities.

The present study examined the potential effects of false alarms. The results indicate that drivers who only receive true alarms moved over more quickly than drivers who receive false alarms. The effect was only detectable in the third event. Furthermore, no difference in move-over time was seen between drivers who received a majority or minority of false alarms. The effect observed in Dingus et al. (1997) and Ben-Yaacov et al. (2002) which stated that when false alarms are in the minority, they will not alter driver behavior was not observed in the current study. It could be that more than three interactions must be included to identify a majority effect. An aspect of false alarm effects that the present study was not able to examine is possible order effects in false alarms. It may be that when first presented with a true alarm, trust is established, and drivers are more likely to comply. Future studies should explore this further.

## Conclusion

The results of the present study indicate that EVA warnings had an effect on encouraging drivers to move over quicker than without warnings. Prior research has suggested that inexperienced drivers would benefit more from EVA warnings. However, no effect of driving experience was found in the present study. The current study included two warning types, one visual and one auditory. No difference between the two modalities was observed.

Previous research suggests that the proportion of false alarms affects compliance. In this study, there were false alarm effects in EVA warnings. The results demonstrate that the presence of false alarms impaired move-over behavior. However, false alarm effects were only observed when comparing drivers who exclusively received true alarms with drivers who received at least one false alarm. No such difference was observed when comparing drivers who received either one or two false alarms.

Future studies should examine the effect of false alarm orders. Ideally, where the everyday interaction with false alarms in cars would be investigated in a more natural setting compared to the method used in the current study.

## Key Points

- Drivers who received an EVA warning moved over more quickly compared to drivers who did not receive an EVA warning.
- Drivers who received true alarms in the first and second EV event moved over more quickly in the third event, compared to drivers who received false alarms in previous events
- Inexperienced and experienced drivers did not significantly differ in the time it took for them to move over for an approaching emergency vehicle.
- No significant difference between an auditory or visual warning modality was observed.
